# First Clinical Results of the Merit WRAPSODY™ Cell-Impermeable Endoprosthesis for Treatment of Access Circuit Stenosis in Haemodialysis Patients

**DOI:** 10.1007/s00270-021-02953-8

**Published:** 2021-09-12

**Authors:** James Gilbert, Jason Rai, David Kingsmore, John Skousen, Nikolaos Ptohis

**Affiliations:** 1 The Oxford Transplant Centre, Churchill Hospital, Old Road, Headington, Oxford, OX3 7LE UK; 2 Queen Elizabeth University Hospital, Glasgow, UK; 3 Merit Medical Systems, Inc, South Jordan, Utah USA; 4 General Hospital of Athens “G.Gennimatas”, Athens, Greece

**Keywords:** Haemodialysis, End-stage renal disease, Stenosis, Occlusion, Arteriovenous access, Arteriovenous fistula, Arteriovenous graft, Stent graft, Covered stent, Endoprosthesis

## Abstract

**Purpose:**

This prospective, observational first in human study evaluated the safety and effectiveness of WRAPSODY^TM^ Cell-impermeable Endoprosthesis (Merit Medical Systems, Inc.) in the treatment of arteriovenous fistula and arteriovenous graft access circuit stenosis.

**Materials and Methods:**

Investigators conducted a prospective analysis of 46 patients with access circuit stenosis from three centres. Treatment sites included the peripheral outflow veins (e.g. cephalic arch, basilic vein swing point; 16 fistula and 10 graft patients); the graft-vein anastomosis (9 patients); and the central veins (up to, but not including the SVC; 11 patients). Primary outcome measures included 30-day freedom from access circuit-related safety events and 30-day target lesion primary patency. Secondary outcome measures included procedural success; device- and procedure-related adverse events; target lesion primary patency; access circuit primary patency; and secondary patency. In-person follow-up was scheduled at 1, 3, 6, and 12 months. An independent data monitoring/clinical event committee adjudicated all reinterventions and device/procedure-relatedness for adverse events.

**Results:**

All initial procedures were successful. All but one patient was free from safety events through the first 30 days (97.8% (45/46)). This event was not device-related. Over the remainder of the study, one adverse event was adjudicated as possibly device-related. Six- and 12-month target lesion primary patency rates were 97.7% (42/43) and 84.6.% (33/39), respectively. Six- and 12-month access circuit primary patency rates were 84.4% (38/45) and 65.9% (29/44), respectively.

**Conclusion:**

Results suggest that the study device is safe and effective for treatment of stenoses in the peripheral and central veins of arteriovenous access circuits.

**Level of Evidence:**

Level 2b, cohort study.

**Supplementary Information:**

The online version contains supplementary material available at 10.1007/s00270-021-02953-8.

## Introduction

End-stage renal disease (ESRD) requiring haemodialysis (HD) is a growing global problem, impacting 554,038 US and 325,180 EU patients in 2018 [1, 2]. HD adequacy is best achieved by creating an arteriovenous access circuit, i.e. an autologous arteriovenous fistula (AVF) or a prosthetic arteriovenous graft (AVG). However, once created the access circuit is prone to complications, leading to problems with dialysis and even access abandonment.

The leading cause of dysfunction is stenosis associated with neointimal hyperplasia and adverse vascular remodelling [3]. Stenosis occurs in all access circuits. Both AVFs and AVGs develop stenoses at key anatomical points including in the draining veins, e.g. the cephalic arch in AVFs, or near the venous anastomosis in AVGs [3]. Both patients with AVFs and AVGs develop central vein stenosis (CVS). While CVS has largely been attributed to prior device use in the central veins, CVS may also develop in patients without prior intervention [4, 5].

Current stenosis treatments are sub-optimal with 12-month patency rates for percutaneous transluminal angioplasty (PTA) below 25% and limited benefit provided by additional bare metal stenting (BMS) [6–14]. Initial trials with drug-coated balloons (DCB) showed promise, though recent trials have questioned their impact [15–18]. Additionally, both PTA and DCB are ineffective when the stenosis is too elastic and recoils, or fibrotic and significant dissection occurs.

Stent grafts (i.e. covered stents or endoprostheses) are recommended for recurrent stenosis due to superior patency rates, reduced reintervention, and financial savings vs. PTA [8–10, 14]. Nevertheless, up to 70% of patients require reintervention within 12 months [9, 19]. Stent graft failure is multi-factorial. The two most established mechanisms are blood interactions with the device surface and edge-stenosis at the device’s inflow and outflow ends [20–22]. A third parallel mechanism was recently identified, namely transmural cell growth through the porous polymer covering leading to neointimal hyperplasia [23].

This article reports the results of a prospective, observational study investigating the safety and effectiveness of WRAPSODY Cell-impermeable Endoprosthesis for the treatment of access circuit stenosis. The study device has been designed with a multilayer, cell-impermeable polymer covering designed to limit restenosis and thrombus formation. The potential benefits of this design were demonstrated in a chronic ovine arterial model, where the study device exhibited reduced stenosis vs. a conventional stent graft [23].

## Materials and Methods

### Study Design

A multicentre, prospective, non-randomised, clinical trial was performed. Forty-six patients with clinically relevant access circuit stenosis were recruited from January 2019 through January 2020. The study was undertaken at three centres experienced with complex vascular access (Churchill Hospital, Oxford, UK; Queen Elizabeth University Hospital, Glasgow, Scotland; General Hospital of Athens “G. Gennimatas”, Athens, Greece). The trial is registered at clinicaltrials.gov (identifier: NCT03644017). The study followed good clinical practice guidelines and local regulatory requirements. An independent data monitoring/clinical event committee (DMC) was formed with three individuals with expertise in clinical trials and safety evaluations. The DMC reviewed and adjudicated reinterventions, access abandonments, as well as device and procedure-relatedness of adverse events.

### Study Population

The eligible population comprised patients undergoing HD through an AV circuit with clinical and radiological evidence of stenosis in the peripheral or central veins. Eligible patients required a mature AVF in the arm with ≥ 1 successful dialysis session or an AVG created ≥ 30 days prior. The target lesion was ≤ 9 cm in length with at least 50% stenosis. Patients with secondary lesions were excluded. Table 1 lists all eligibility criteria.

**Table 1:** Inclusion and exclusion criteria

Inclusion criteria	
1	Patient has signed informed consent
2	Patient ≥ 21 years old
3	Patient is undergoing chronic haemodialysis or other forms of renal replacement therapy including transplantation and has one of the following being used: a. AVG placed in the arm ≥ 30 days prior OR b. Mature AVF in the arm with at least one successful dialysis session completed
4	Angiographic evidence of (multiple stenoses may exist within the target lesion):
	a. Lesion ≤ 9 cm in length in arm or thoracic central vein, not located within the needling segment of AVF, and ends before the superior vena cava, OR
	b. Lesion ≤ 9 cm in length in arm or thoracic central vein, not located within the needling segment of an AVG, and ends before the superior vena cava
5	The target lesion has ≥ 50% stenosis
6	Patient has clinical or haemodynamic evidence of venous outflow stenosis or obstruction
7	Full expansion of an appropriately sized standard angioplasty balloon (in the investigator’s opinion) has been achieved during primary angioplasty at the target lesion prior to enrollment

Exclusion criteria	
1	Patient has undergone a surgical intervention of AVF/AVG ≤ 30 days from the date of the initial study procedure
2	Patient has a previous stent or stent graft placed in the venous outflow circuit ≤ 30 days from the date of the initial study
3	Active haemodialysis access is not in the arm
4	A pseudoaneurysm is present within the target lesion
5	Target lesion is: a. In the superior vena cava
	b. In the jugular vein
	c. Under the clavicle
	d. Requires stent graft placement across the elbow
	e. In the needling segment of an AVF or AVG anastomosis
	f. Located within a stent
6	Lesions, other than the target lesion, in the venous outflow circuit with > 30% stenosis. Note that patients with secondary lesions may be included IF the lesions have been treated > 30 days before study procedure AND have less than 30% residual stenosis
7	Known or suspected infection of the haemodialysis access site and/or septicemia
8	Permanent pacemaker or automated implantable cardioverter defibrillator (AICD) on the side with the target lesion
9	Current central venous catheter for dialysis access
10	Uncorrectable coagulation disorders
11	Hypersensitivity to nickel titanium alloy
12	Patient is enrolled in another investigational study
13	Patient is unable or unwilling to comply with the protocol requirements
14	Life expectancy ≤ 12 months
15	Patient cannot receive heparin or equivalent anticoagulant
16	Allergy to radiographic contrast material which cannot be adequately premedicated
17	Patient is pregnant, breast feeding, or pre-menopausal and intending to become pregnant
18	Patient’s access is anticipated to be abandoned within 3 months
19	Patient has a thoracic central vein obstruction that would lead to stent graft placement across the internal jugular vein
20	Patient’s haemodialysis access is thrombosed
21	Active malignancy other than non-melanomatous skin cancer
22	Any other condition deemed exclusionary in the opinion of the investigator

**Fig. 1:**
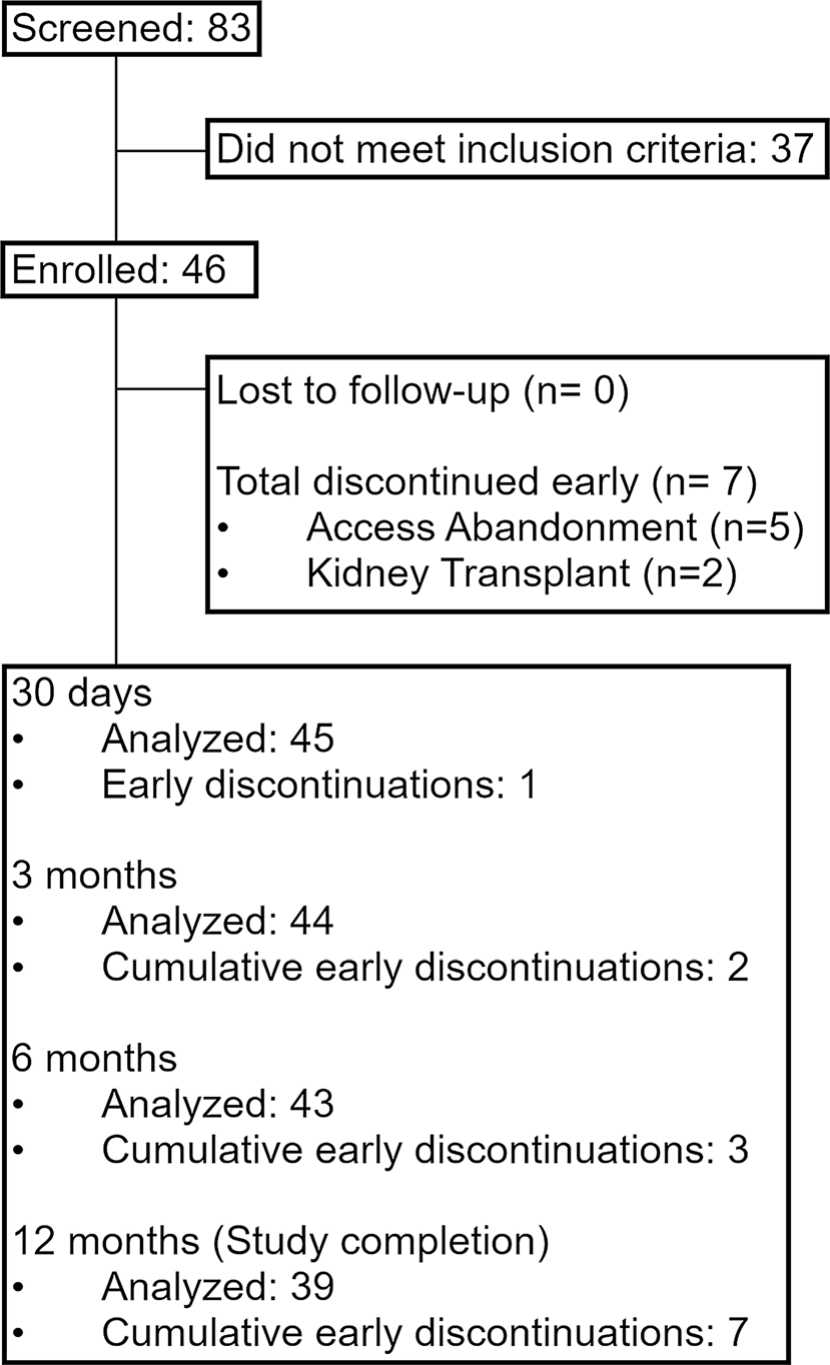
Flow diagram showing patient enrolment and retention

All patients provided written informed consent and were informed that they had the right to withdraw from the study at any time for any reason. Eighty-three patients were consented with 46 meeting the eligibility criteria and receiving treatment (consecutive enrolment). Enrolled patients included 16 patients with AVF and 10 with AVG and stenosis of the peripheral outflow veins (e.g. cephalic arch, basilic vein swing point); 9 with stenosis at the graft-vein anastomosis; and 11 with CVS. Figure 1 summarizes patient accountability. Tables 2 and 3 detail patient demographics and access circuit/target lesion characteristics. Tables S1 and S2 list patient’s concurrent medical conditions and anticoagulant/antiplatelet regimens.

**Table 2:** Baseline demographics of haemodialysis patients by access type and lesion location

	AVF peripheral	AVG anastomosis	AVG peripheral	AVF/AVG central	All patients
	*N* = 16	*N* = 9	*N* = 10	*N* = 11	*N* = 46
Age—% (*n*)					
< 65	50% (8)	33% (3)	40% (4)	45% (5)	43% (20)
65–74	13% (2)	22% (2)	40% (4)	27% (3)	24% (11)
≥75	38% (6)	44% (4)	20% (2)	27% (3)	33% (15)
Sex—M:F	6:10	6:3	2:8	8:3	22:24
BMI—Mean (SD)	27 (7.5)	30 (4.8)	27 (5.0)	29 (7.8)	28 (6.6)
Co-Morbidity—% (*n*)					
Diabetes mellitus	38% (6)	67% (6)	20% (2)	27% (3)	37% (17)
Dyslipidemia	19% (3)	-	40% (4)	45% (5)	26% (12)
Hypertension	69% (11)	56% (5)	20% (2)	36% (4)	48% (22)
Smoking	38% (6)	33% (3)	40% (4)	64% (7)	43% (20)

**Table 3:** Access circuit and target lesion characteristics of haemodialysis patients

	AVF peripheral	AVG anastomosis	AVG peripheral	AVF/AVG central	All patients
	*N* = 16	*N* = 9	*N* = 10	*N* = 11	*N* = 46
*Access circuit characteristics*					
AVF location	16	–	–	8	24
Radiocephalic	12.5% (2)	–	–	0	8.3% (2)
Brachiocephalic	75.0% (12)	–	–	100% (8)	83.3% (20)
Brachialbasilic	12.5% (2)	–	–	0	8.3% (2)
AVG Arterial Anastomosis Location	–	9	10	3	22
Brachial	–	77.8% (7)	100% (10)	100% (3)	90.9% (20)
Radial	–	11.1% (1)	0	0	4.5% (1)
Other	–	11.1% (1)	0	0	4.5% (1)
AVG Venous Anastomosis Location	–	9	10	3	22
Axillary	–	22.2% (2)	30.0% (3)	0	22.7% (5)
Basilic	–	11.1% (1)	20.0% (2)	0	13.6% (3)
Brachial	–	0	30.0% (3)	0	13.6% (3)
Cephalic	–	55.6% (5)	20.0% (2)	100% (3)	45.5% (10)
Other	–	11.1% (1)	0	0	4.5% (1)
*Target Lesion Characteristics*					
*Target Lesion Location*					
Subclavian	–	–	–	27.3% (3)^1^	6.5% (3)^1^
Brachiocephalic	–	–	–	81.8% (9)^1^	19.6% (9)^1^
Axillary	0	–	30.0% (3)	–	6.5% (3)
Cephalic Vein Arch	62.5% (10)^2^	–	0	–	21.7% (10)^2^
Cephalic Vein Outflow	18.8% (3)^2^	–	10.0% (1)	–	8.7% (4)^2^
Brachial	12.5% (2)	–	60.0% (6)	–	17.4% (8)
Basilic	12.5% (2)	–	0	–	4.3% (2)
Venous Anastomosis	–	100% (9)	–	–	19.6% (9)
*Target lesion—mean (SD)*					
Diameter (mm)	7.5 (2.0)	6.0 (0.9)	6.6 (1.0)		7.8 (2.3)
Mean length (mm)	36.3 (16.5)	33.8 (23.8)	39.2 (19.3)	26.9 (8.9)	34.2 (17.4)
Mean stenosis %	68.3% (14.0)	75.3% (8.3)	70.9% (4.9)	69.7% (12.2)	70.6% (11.1)
De Novo Lesion	31.3% (5)	55.6% (5)	80.0% (8)	54.5% (6)	52.2% (24)

1 One patient had a target lesion that extended from the subclavian into the brachiocephalic vein and is double counted

2 One patient had a target lesion spanning the cephalic vein arch and cephalic vein outflow and is double counted

### Study Device

**Fig. 2:**
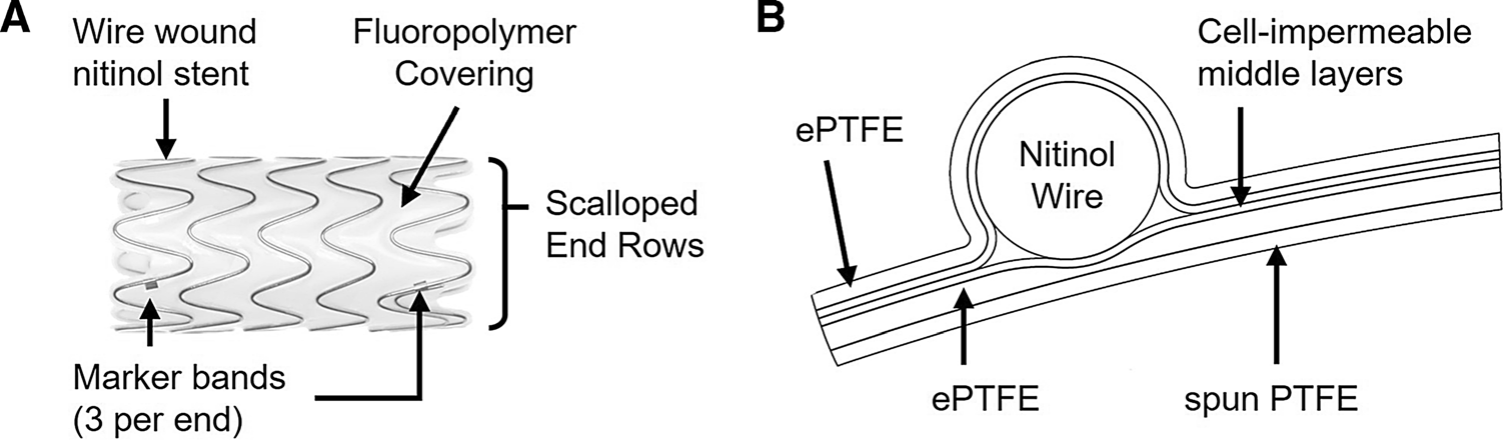
Study device. (**A**) Representative image of a 14 mm diameter x 30 mm device. (**B**) Cross-sectional schematic surrounding a single nitinol wire illustrating the organization of the multilayer graft covering. Similar to other stent grafts, the abluminal layer is composed of ePTFE to facilitate cell-ingrowth to anchor the device in place. Directly adjacent to the nitinol wire are layers of cell-impermeable fluoropolymer to prevent transmural cell migration through the graft covering. Below the innermost cell-impermeable layer is a second layer of porous ePTFE. The luminal surface of the graft is composed of a spun PTFE designed to reduce fibrin deposition and subsequent thrombus formation

WRAPSODY Endoprosthesis (Merit Medical Systems, Inc.; South Jordan, Utah, USA) is a self-expanding, cell-impermeable endoprosthesis. The endoprosthesis is composed of a helically wound nitinol wire stent fully encapsulated in a multilayer fluoropolymer covering (Fig. 2). The covering’s luminal layer is composed of a spun polytetrafluoroethylene (spun PTFE) designed to limit fibrin deposition and thrombus formation. The cell-impermeable middle layer is designed to prevent transmural cell growth. The outer layer is made of a traditional expanded polytetrafluoroethylene (ePTFE), permitting cell-ingrowth for device anchoring. The device is offered in various sizes, ranging in diameter from 6.0–16.0 mm (allowing treatment of vessels from 4.6–14.4 mm in diameter) and ranging in length from 30−125 mm.

### Study Treatment and Follow-Up

Procedures were undertaken by four operators across the three sites, all with experience in interventional procedures. An uncoated standard angioplasty balloon (type and size left to site discretion) was used for pre-dilation. Full expansion of the pre-dilation balloon was required. Following pre-dilation, reassessment, and eligibility confirmation, patients were treated with the study device. To ensure adequate fixation and wall vessel apposition, devices were deployed in a 10–25% oversized configuration (vs. adjacent healthy vessel) with at least 1 cm overlap with healthy vessel or the synthetic graft. Devices were post-dilated with a balloon no greater than the endoprosthesis’ diameter. If multiple devices were deployed in an overlapped configuration, post-dilation of the first device occurred before deploying the second. If different sized devices were overlapped to accommodate vessel diameter changes, the smaller device was deployed first. Patients were treated and discharged according to each centre’s standard of care, including anticoagulant or antiplatelet administration. Table S4 summarizes device disposition.

An in-person physical examination was scheduled at 1, 3, 6, and 12 months post-procedure with additional visits as needed for assessment and treatment of access circuit dysfunction. Follow-up times were chosen to facilitate analysis over the critical period when current interventions fail. Physical examinations included at minimum an assessment of hand, arm, neck, and trunk oedema; pain related to dialysis circuit; respiratory and neurological symptoms; skin changes; and a review of AEs and interventions. The access circuit was also assessed for quality of flow.

### Outcome Measures

The primary safety measure was the proportion of patients without any localised or systemic safety events affecting the access circuit during the first 30 days that resulted in surgery, hospitalization, or death. The primary effectiveness measure was the target lesion primary patency rate (TLPP) at 30 days. TLPP was defined as the interval of uninterrupted patency from initial study procedure to the next intervention performed on the target lesion or uncorrectable target lesion occlusion, whichever occurs first.

Secondary outcome measures included clinical (resumption of successful dialysis for at least one session), anatomical (less than 30% residual stenosis), and procedural success (achievement of both clinical and anatomical success). Device- or procedure-related adverse events and TLPP were further assessed at 3, 6, and 12 months. Assisted TLPP, access circuit primary patency (ACPP), and access circuit secondary patency were evaluated at 1, 3, 6, and 12 months. Table S4 contains a definition for each outcome measure.

### Statistical Methods

Statistics and Data Corporation (SDC) and Clinlogix provided statistical analysis services for the study. Statistics were calculated using SAS (v9.4, SAS Institute Inc.). As this was a single-arm study, statistical analysis only included descriptive statistics (e.g. mean and standard deviation). Patency values were calculated using the Kaplan–Meier method. An interim analysis was preformed when all patients completed the 6-month visit.

### Role of the Study Sponsor

Study devices were supplied by the sponsor. The sponsor was involved in establishing the study design; data collection, analysis, and interpretation; as well as the preparation of and decision to submit the manuscript. All authors confirm that they had full access to the study data and accept responsibility for publication submission.

## Results

### Procedural Outcomes

**Fig. 3:**
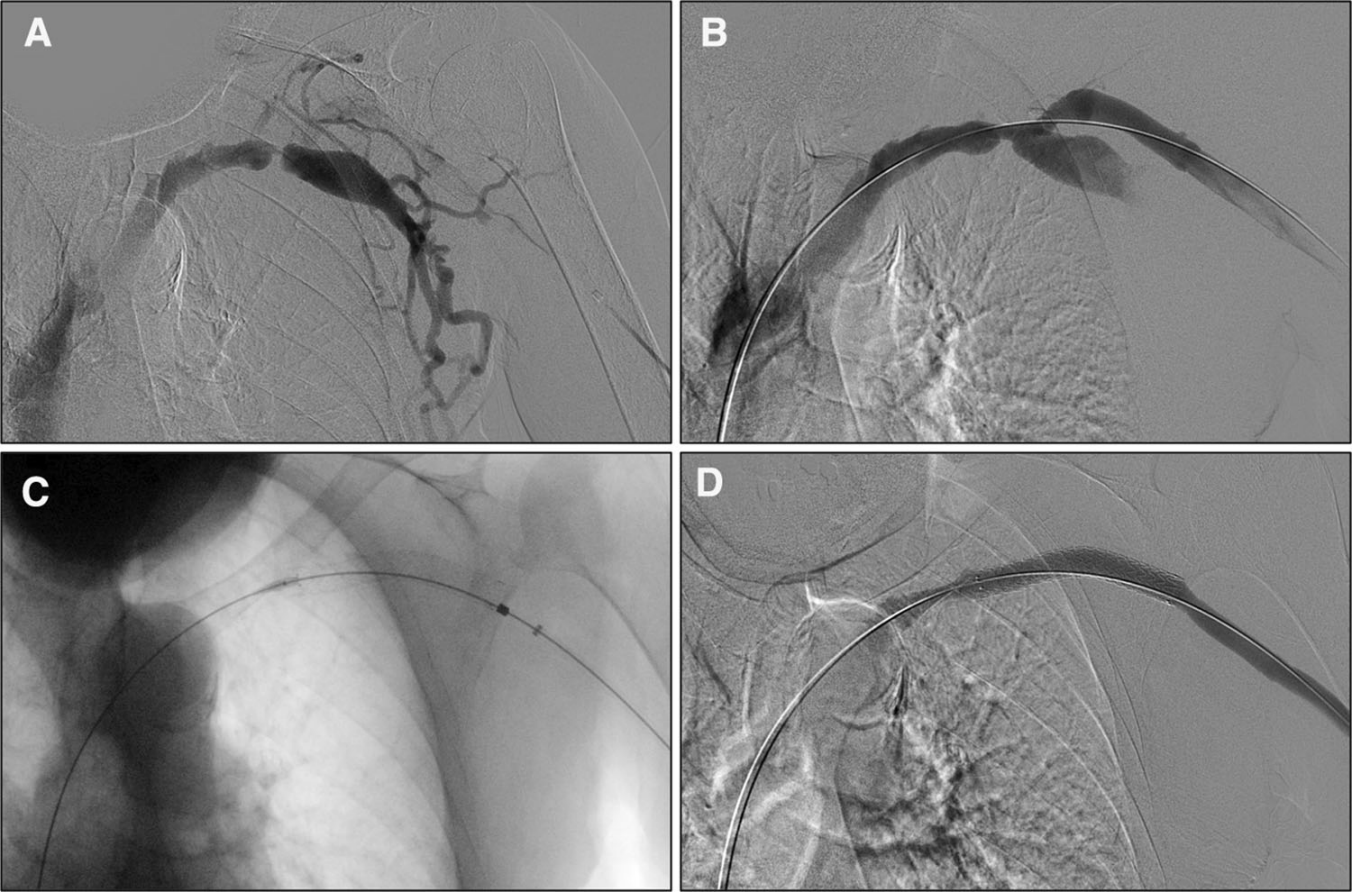
Example procedural outcomes. Treatment of a patient with a left brachiocephalic AVF that presented with a stenosis at the cephalic arch extending into the distal subclavian vein. (**A** & **B**) Fistulograms taken (**A**) preintervention and (**B**) after pre-dilitation of the stenosis. (**C**) Fluoroscopy of fully deployed study device. (**D**) Completion fistulogram

Anatomical, clinical, and procedural success were achieved in all cases (100% [46/46]). Figure 3 highlights the procedural outcomes of an example case.

### Safety Outcomes

One patient required surgery within 30 days of the study procedure following AVF circuit thrombosis (primary safety of 97.8% [45/46]). As reported by the site and confirmed by the DMC, this event was related to a pre-existing perianastomotic seroma causing extrinsic compression near the inflow end of the circuit and not related to the study procedure or device. With this event, there were 47 AEs in a total of 16 patients and 17 serious adverse events (SAEs) in 10 patients. None were unanticipated adverse device effects. All events followed expectations for the patients enrolled.

There was one AE, a thrombosed fistula, where the access circuit was not salvageable and there was no imaging to assess device-relatedness. Therefore, this event was listed as possibly device- and procedure-related (Table 4). Four other AEs were adjudicated by the DMC to be only procedure-related. Of note, one AVG peripheral patient experienced brachial vein rupture at the vascular access site. The rupture was successfully treated via balloon tamponade and placement of additional non-study stent grafts. One central patient experienced migration of two overlapped devices during the index procedure. The investigator, DMC, and an independent physician review (following DMC recommendation) adjudicated that migration was the result of inappropriate vessel sizing during pre-dilation (oversized pre-dilation balloon was used to assess vessel diameter) leading to the placement of inappropriately sized study devices. Specifically, the devices were excessively oversized for the vessel at the peripheral aspect of the stents, in contraindication to the IFU. This in combination with malapposition to the vessel wall along the central aspect of the device led to the migration. The devices were repositioned to the inferior vena cava where they were believed to be well seated and two additional, appropriately sized devices were placed to treat the original stenosis. Upon further assessment at the 30-day visit, it was observed that the initial two devices had migrated again (likely due to inadequate wall apposition during Valsalva manoeuver). To ensure against any further migration, the devices were pulled to the iliac vein and secured with an additional high radial force bare metal stent. No further migration issues occurred in either the repositioned devices or the replacement devices placed at the target lesion.

**Table 4:** Device-and procedure-related events as adjudicated by DMC

Patient ID	Event description	Device-relatedness	Procedure-relatedness
AVF peripheral			
302-002	Mild steal syndrome	None	Probable
AVG anastomosis			
-	–	–	–
AVG peripheral			
301-031	Brachial vein rupture	None	Definite
AVF/AVG central			
301-048	Migration of stent grafts	None	Definite
301-048	Migration of previously repositioned stent grafts	None	Definite
302-020	Thrombosed fistula	Possible*	Possible

^*^ Listed as possibly device-related as the access circuit was not salvageable so there was no imaging to assess device-relatedness

### Performance Outcomes

**Fig. 4:**
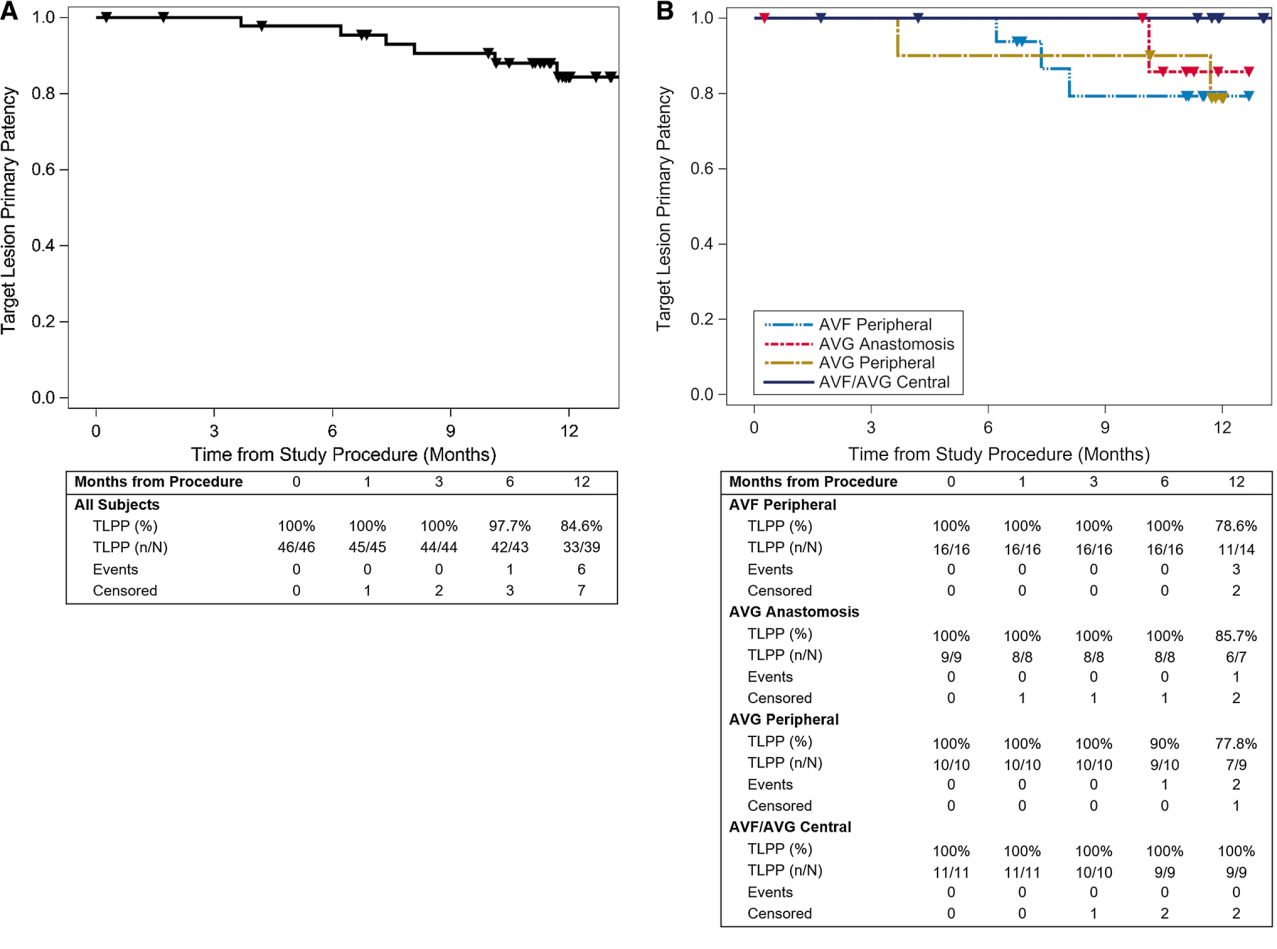
TLPP through 12 months. Kaplan–Meier (KM) curves for (**A**) all patients and (**B**) sub-cohorts based on access type and lesion location. The tables below each graph list the number of patients at risk, those censored (i.e. exited the study for renal transplant or loss to follow-up), and those who experienced an event leading to the loss of TLPP. Triangles on the KM curves represent either a patient’s date of censoring or final follow-up visit

**Fig. 5:**
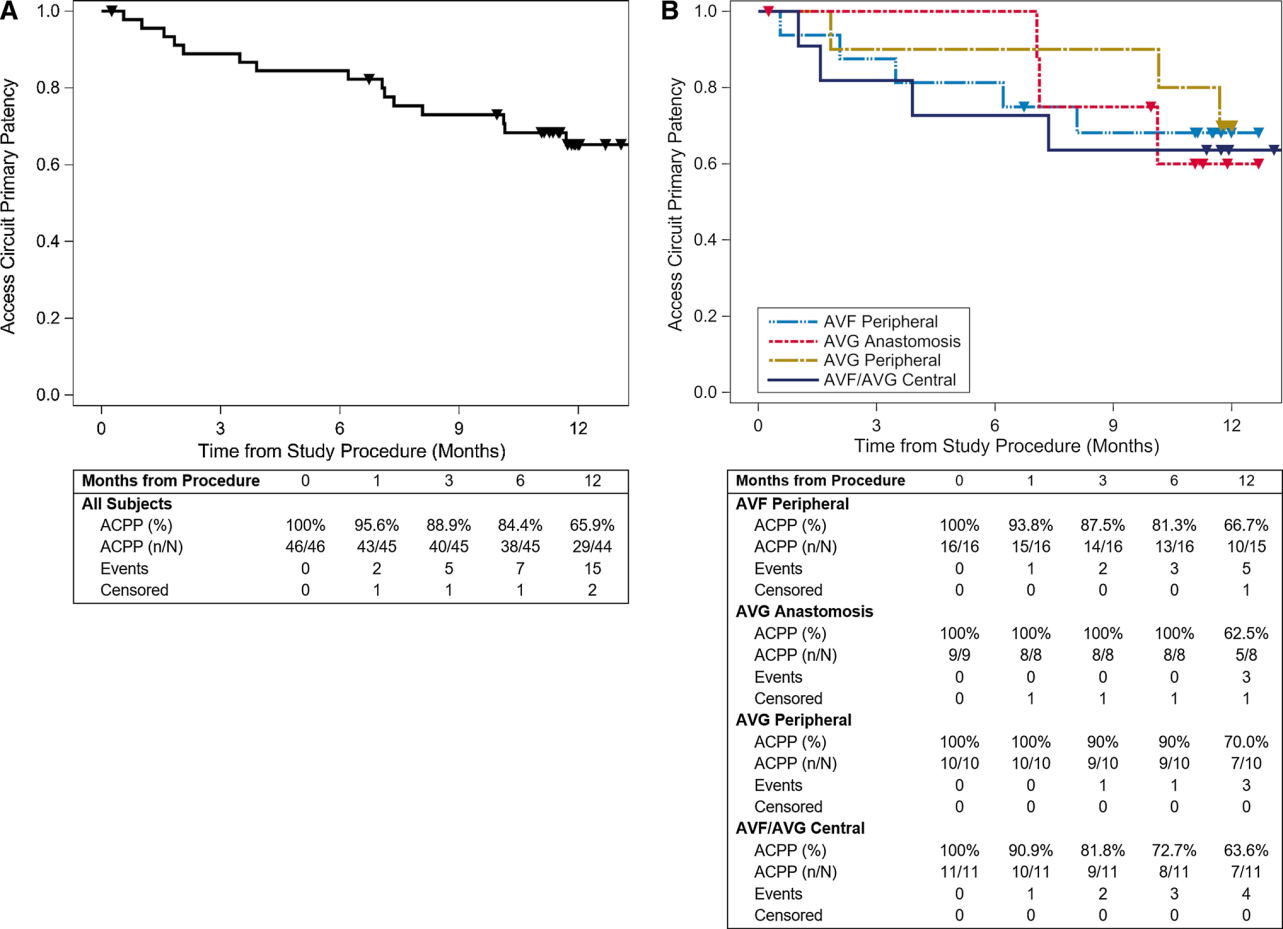
Kaplan–Meier curves for access circuit primary patency (ACPP). ACPP through 12 months. Kaplan–Meier (KM) curves for (**A**) all patients and (**B**) sub-cohorts based on access type and lesion location. The tables below each graph list the number of patients at risk, those censored (i.e. exited the study for renal transplant or loss to follow-up), and those who experienced an event leading to the loss of ACPP. Triangles on the KM curves represent either a patient’s date of censoring or final follow-up visit

One patient underwent kidney transplant 7 days after the index procedure and was excluded from all patency calculations. No reinterventions were required to maintain target lesion patency through 30 days (primary effectives of 100% [45/45], Fig. 4a). Six-month TLPP was 97.7% (42/43) and 84.6% (33/39) at 12 months (Fig. 4a). TLPP was similar irrespective of access type and lesion location (Fig. 4b) or whether patients were treated with a single device or multiple overlapped devices (Table 5). Reintervention at the target lesion was successful in maintaining patency in all cases (12-month assisted TLPP: 100% [39/39]). ACPP was 84.4% (38/45) at 6 months and 65.9% (29/44) at 12 months (Fig. 5). Access circuit secondary patency was 88.6% (39/44) at 12 months (Table 6).

**Table 5:** Target lesion primary patency (TLPP) for patients treated with a single or multiple overlapped devices

		AVF peripheral % (n/N)	AVG anastomosis % (n/N)	AVG peripheral % (n/N)	AVF/AVG central % (n/N)	All patients % (n/N)
6 months	Single device	100% (11/11)	100% (4/4)	90.0% (9/10)	100% (6/6)	96.8% (30/31)
	Overlapped	100% (5/5)	100% (4/4)	–	100% (3/3)	100% (12/12)
12 months	Single device	77.8% (7/9)	100% (3/3)	77.8% (7/9)	100% (6/6)	85.2% (23/27)
	Overlapped	80.0% (4/5)	75.0% (3/4)	–	100% (3/3)	83.3% (10/12)

**Table 6:** Secondary patency of the access circuit

	AVF peripheral % (n/N)	AVG anastomosis % (n/N)	AVG peripheral % (n/N)	AVF/AVG central % (n/N)	All patients % (n/N)
30 Days	100% (16/16)	100% (8/8)	100% (10/10)	100% (11/11)	100% (45/45)
3 months	100% (16/16)	100% (8/8)	100% (10/10)	90.9% (10/11)	97.8% (44/45)
6 months	100% (16/16)	100% (8/8)	100% (10/10)	81.8% (9/11)	95.6% (43/45)
12 months	93.3% (14/15)	87.5% (7/8)	90.0% (9/10)	81.8% (9/11)	88.6% (39/44)

## Discussion

Results indicate that the study device can safely and effectively treat stenoses in the peripheral outflow and central veins of AV access circuits. Only one safety event (unrelated to the study device or procedure) occurred within 30 days of treatment and only one AE was adjudicated as possibly study device-related. The 6- and 12-month TLPP and ACPP rates were unexpectedly high. While needing further confirmation, the study device’s design may be a contributing factor in limiting restenosis. If confirmed in larger studies, this degree of patency preservation should reduce revision frequency and access abandonment rates, leading to less hospitalisation and lower healthcare costs [24, 25].

The high patency rates were seen across all access types and lesion locations. While not a comparative study, performance in patients with a peripheral stenosis in their AVF access circuit and in patients with stenosis of the graft-vein anastomosis compares favourably to the results from previous large-scale studies that have investigated other stent grafts for these uses [7, 9, 19, 26–28]. Furthermore, current results suggest that the device can safely treat CVS in the subclavian and brachiocephalic veins. Though care should always be given when using a stent graft in the central veins to avoid damaging the device due to external compression from the clavicle or jailing important collaterals.

Patency rates also compare favourably to historic results for other treatments (i.e. PTA, BMS, and DCB). However, it must be remembered that stent graft use may not be universally appropriate. One should consider the potential for and impact of jailing collateral vessels, restricting future access sites, or compromising device integrity (via compression, flexion, or puncture). Furthermore, appropriate vessel sizing is critical to avoid vessel damage and to ensure against device migration.

The high patency results vs. historical results for other devices may be attributed to a variety of factors. A previous preclinical study showed that the study device had a reduced stenosis rate than a conventional stent graft [23]. Therefore, these findings may be due to the study device’s underlying stent mechanics and multilayer graft covering. Beyond device design, differences in eligibility criteria may also underlie the current high patency results. This study excluded patients with thrombus or secondary lesions in the access circuit and other studies have noted a marked patency rate reduction when patients present with either condition [26–28]. This exclusion was intentional in order to avoid other variables confounding the assessment of safety and access circuit outcomes that might be associated with the novel aspects of the study device. Nevertheless, the lack of patients with thrombus or secondary lesions does not appear to fully explain patency rate differences with previous studies. Specifically, the respective 6-month TLPP rates for patients without thrombus or secondary lesions from these previous studies ranged from 64.6–76.9% and 57.0– 79.3% while the ACPP rates were 42.3–49.7% and 45.1–59.8% [26–28].

Beyond the stringent eligibility criteria, another limitation is the study’s size and lack of a control. To address these limitations, a larger, multicentre study, The Merit WRAPSODY AV Access Efficacy Study (NCT04540302), has been initiated. The study also enrolled patients with AVF and AVG access with a variety of commonly occurring lesions impacting these access circuits. While this design facilitated an initial screening of potential issues across the array of haemodialysis patients, case heterogeneity also limited the conclusions that can be drawn regarding specific situations. Other limitations include lack of a standardized measurement procedure and image assessment by an independent angiographic core laboratory. The current study also left use of anticoagulants and antiplatelets to the discretion of each site, creating the potential for site-to-site variability.

## Conclusion

Current results indicate that the study device may represent a safe and effective treatment for AV access circuit stenoses. The high patency rates are promising and may offer patients with access circuit dysfunction improved treatment with more durable outcomes. Additional follow-up in larger RCTs is needed to verify these results.

## Supplementary Information

Below is the link to the electronic supplementary material.

Supplementary file1 (DOCX 19 KB)
