# Supplementary material for: First Clinical Results of the Merit WRAPSODY™ Cell-Impermeable Endoprosthesis for Treatment of Access Circuit Stenosis in Haemodialysis Patients
Source: Cardiovasc Intervent Radiol. 2021 Sep 12;44(12):1903–13. doi: 10.1007/s00270-021-02953-8 (PMC8626397; doi:10.1007/s00270-021-02953-8)
Supplement: Supplementary file 1 — Supplementary file1 (DOCX 19 KB) [file 270_2021_2953_MOESM1_ESM.docx]

# Supplementary Tables

## **Table S1: Concurrent medical conditions of haemodialysis patients with access circuit stenosis.**

|  | All patients |
| --- | --- |
|  | N = 46 |
| Anaemia | 18 (39%) |
| Cardiac disorders | 12 (26%) |
| Congenital cystic kidney disease | 1 (2%) |
| Ear and labyrinth disorders | 2 (4%) |
| Endocrine disorders | 14 (30%) |
| Glaucoma | 1 (2%) |
| Gastrointestinal disorders | 2 (4%) |
| Hepatic failure | 1 (2%) |
| Hypertryglyceridemia | 1 (2%) |
| Hyperuricemia | 2 (4%) |
| Infections and infestations | 2 (4%) |
| Metabolism and nutrition disorders | 28 (61%) |
| Musculoskeletal and connective tissue disorders | 3 (7%) |
| Uterine leiomyoma | 1 (2%) |
| Nervous system disorders | 5 (11%) |
| Psychiatric disorders | 8 (17%) |
| Renal and urinary disorders | 41 (89%) |
| Reproductive and breast disorders | 2 (4%) |
| Respiratory, thoracic and mediastinal disorders | 4 (9%) |
| Skin and subcutaneous tissue disorders | 1 (2%) |
| Surgical and medical procedures | 4 (9%) |
| Vascular disorders | 22 (48%) |

## **Table S2: Concurrent antiplatelet and anticoagulant taken by haemodialysis patients.**

|  | All Patients |
| --- | --- |
|  | N = 46 |
| Platelet aggregation inhibitor |  |
| - Salospir | 8 |
| - Aspirin | 7 |
| - Plavix | 4 |
| - Clopidogrel | 3 |
| - Clovelen | 1 |
| - Globel | 1 |
| Heparin group |  |
| - Tinzaparin | 14 |
| - Dalteparin | 4 |
| - Enoxaparin | 2 |
| - Heparin | 2 |
| Vitamin K antagonist |  |
| - Warfarin | 7 |
| - Sintrom | 3 |
| Direct Factor Xa inhibitor |  |
| - Eliquis | 1 |
| Patients on multi-drug regimen | 13 |

## **Table S3: Dispensation of stent grafts used to treat access circuit stenosis.**

|  | AVF  Peripheral | AVG Anastomosis | AVG  Peripheral | AVF/AVG Central | All  Patients |
| --- | --- | --- | --- | --- | --- |
|  | N=22 devices | N=13 devices | N=10 devices | N=17 devices | N=62 devices |
| 6 mm x 50 mm | 1 | 1 | 3 | - | 5 |
| 6 mm x 75 mm | - | 2 | 1 | - | 3 |
| 7 mm x 50 mm | 2 | 5 | 2 | - | 9 |
| 7 mm x 75 mm | 2 | - | 1 | - | 3 |
| 7 mm x 100 mm | - | 1 | - | - | 1 |
| 8 mm x 50 mm | 4 | 1 | 2 | - | 7 |
| 8 mm x 75 mm | 4 | 1 | 1 | - | 6 |
| 8 mm x 100 mm | 1 | - | - | - | 1 |
| 9 mm x 50 mm | 1 | 1 | - | 1 | 3 |
| 9 mm x 75 mm | 1 | 1 | - | 1 | 3 |
| 10 mm x 50 mm | 2 | - | - | 4 | 6 |
| 10 mm x 75 mm | 3 | - | - | - | 3 |
| 12 mm x 40 mm | 1 | - | - | 4 | 5 |
| 14 mm x 30 mm | - | - | - | 1 | 1 |
| 14 mm x 40 mm | - | - | - | 4 | 4 |
| 16 mm x 40 mm | - | - | - | 2 | 2 |
| Patients with Multiple Devices  % (n/N patients) | 31% (5/16) | 44% (4/9) | 0% (0/10) | 36% (4/11) | 28% (13/46) |

## **Table S4: Outcome measure definitions.**

| Outcome Measure | Definition |
| --- | --- |
| Clinical success | Resumption of successful dialysis through existing access for at least one session following initial study procedure. |
| Anatomic success | Less than 30% residual stenosis immediately following study procedure, based on physician’s assessment of the completion fistulogram. |
| Procedural success | The achievement of both clinical and anatomic success. |
| Safety | Proportion of patients without any localized or systemic safety events through 30 days that affect the access or venous outflow circuit and resulted in surgery, hospitalization, or death. Safety events in this calculation did not include venous outflow obstructions, which are captured in the calculation of Assisted target lesion primary patency and secondary patency of the venous outflow circuit. |
| Target Lesion Primary Patency (TLPP) | Interval of uninterrupted patency from initial study procedure to the next intervention performed on the target lesion or uncorrectable target lesion occlusion, whichever occurs first. |
| Assisted TLPP | Time following study procedure until uncorrectable target lesion occlusion. |
| Access Circuit Primary Patency (ACPP) | Time following initial study procedure until the next venous outflow circuit intervention (including interventions to the target lesion) or complete access abandonment, whichever occurs first. |
| Access Circuit Secondary Patency | Time following initial study procedure until complete access abandonment. |
